# A method to generate small-scale, high-resolution sedimentary bedform architecture models representing realistic geologic facies

**DOI:** 10.1038/s41598-017-09065-9

**Published:** 2017-08-23

**Authors:** T. A. Meckel, L. Trevisan, P. G. Krishnamurthy

**Affiliations:** 1 Bureau of Economic Geology, Jackson School of Geosciences, The University of Texas at Austin, Austin, TX USA; 2 Department of Petroleum and Geosystems Engineering, The University of Texas at Austin, Austin, TX USA

## Abstract

Small-scale (mm to m) sedimentary structures (e.g. ripple lamination, cross-bedding) have received a great deal of attention in sedimentary geology. The influence of depositional heterogeneity on subsurface fluid flow is now widely recognized, but incorporating these features in physically-rational bedform models at various scales remains problematic. The current investigation expands the capability of an existing set of open-source codes, allowing generation of high-resolution 3D bedform architecture models. The implemented modifications enable the generation of 3D digital models consisting of laminae and matrix (binary field) with characteristic depositional architecture. The binary model is then populated with petrophysical properties using a textural approach for additional analysis such as statistical characterization, property upscaling, and single and multiphase fluid flow simulation. One example binary model with corresponding threshold capillary pressure field and the scripts used to generate them are provided, but the approach can be used to generate dozens of previously documented common facies models and a variety of property assignments. An application using the example model is presented simulating buoyant fluid (CO_2_) migration and resulting saturation distribution.

## Introduction

Two longstanding challenges in flow and transport problems have been the accurate representation of geologic heterogeneity in numerical models and quantifying the influence of that heterogeneity on fluid flow^1,2^. Here heterogeneity refers to mm- to m-scale variability in grain sizes and architecture (spatial organization) associated with clastic depositional processes as manifested in descriptive structures. There is a long history of study of depositional sedimentary structures at these scales and their influence on fluid flow has been known for decades^3–7^. Critical aspects of geological heterogeneity related to small-scale depositional features have been documented for a variety of considerations^8–13^. These types of studies highlight the importance of understanding the influence of depositional heterogeneity at relatively small scales and the challenges involved in adequately representing such heterogeneity for various applications.

The digital models presented here are a variant of traditional geostatistical representations of depositional facies^1,14^, which many geoscientists agree were a vast improvement over homogeneous/isotropic representations and quite advantageous, but often fall short of effectively representing the small scale details in their most recognizable and descriptive forms. The method for generating bedform architecture models (BAM) presented here is most closely associated with geometric approaches^15,16^ in that the models are meant to be geologically plausible, depositionally realistic, and visually satisfying from a geologic perspective. The focus is on relating the depositional processes and resulting characteristic descriptive architecture to the distribution of material properties (i.e. grain size, permeability, porosity, threshold capillary pressure). Geostatistical techniques are often used for populating large-scale models for applications such as reservoir flow simulation^17^ and hydrogeological applications^18^. While geostatistical techniques continue to evolve in complexity^19^, their ability to generate depositionally realistic small-scale representations of standard geologic features remains a challenge. The approach here differs from the sedimentary process forward model approach that requires complex computational fluid dynamics, including turbulence^20^. Other attempts at representing sedimentary architecture at small scale include utilization of natural specimens or outcrops in two and three dimensions^21–24^.

**Figure 1:**
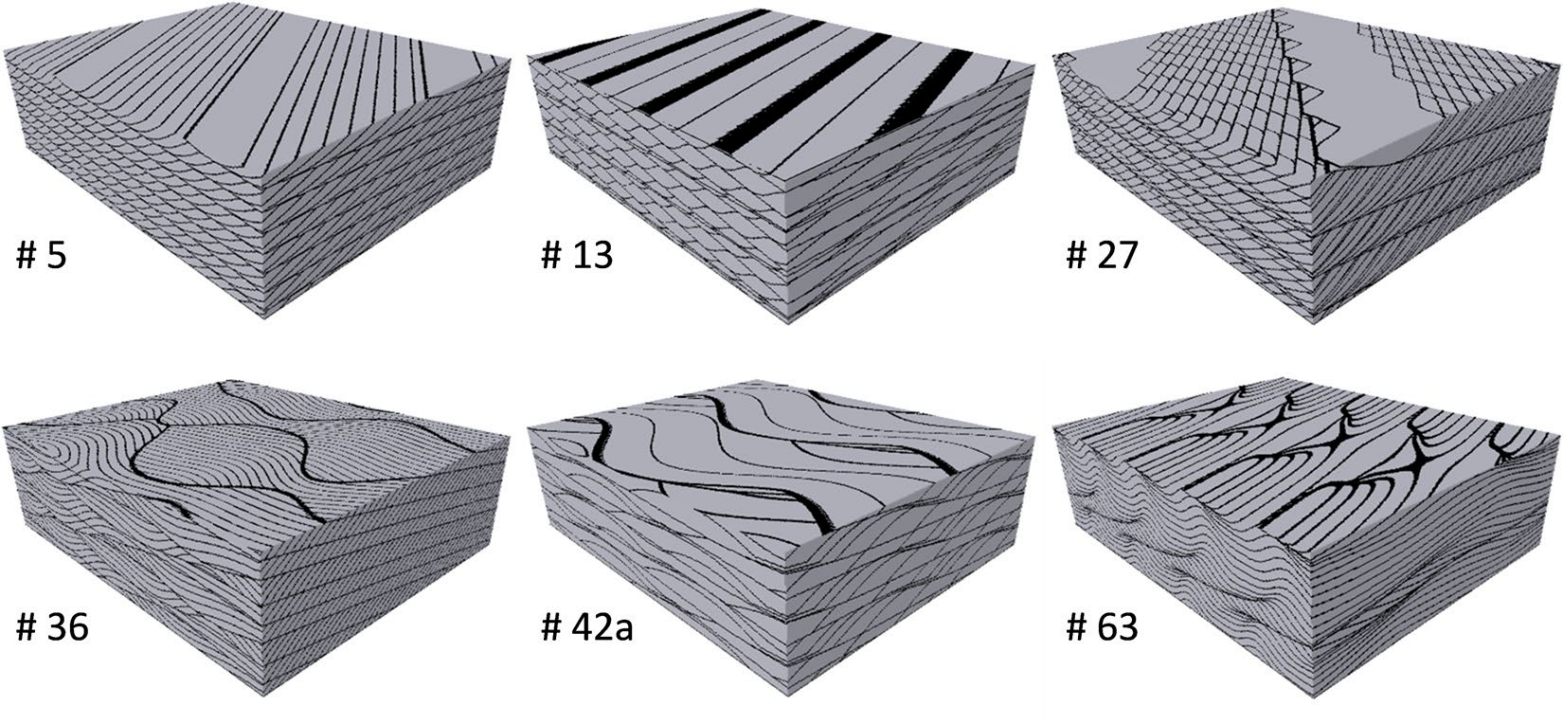
Six examples of 3D bedform architecture models (BAM) with associated numbers for specific figures from Rubin and Carter^26^. Black lines represent laminae that define bedforms with characteristic descriptive and process-based understanding. Horizontal and vertical scales are user-defined, but typically 10’s of centimeters.

The approach presented here extends a well-documented and widely utilized set of codes originally presented by Rubin^25^ and later published by Rubin and Carter^26^ as a set of MATLAB routines. David Rubin pioneered the digital representation of clastic depositional features, linking our understanding of sediment transport processes, bedform morphology, and descriptive sedimentology. The ability to generate bedform images and visualize bedform deposition and migration represented a major advancement in sedimentary geology and has been utilized by sedimentologists and stratigraphers to understand sedimentary processes and to interpret structures observed in the field and subsurface cores^27^. The original Rubin^25^ computer program was based on a geometric model, using sine curves to generate surfaces that mimic well-documented bedforms. The numerical modification of up to three sine curves simulated the migration and erosion of bedforms, defined by 75 user-definable geometric parameters^26^. While this approach does not mimic hydrodynamic processes related to deposition, it does produce readily recognizable features (i.e. similar to outcrop and modern environments) that can be related to depositional processes. The original output consists of three-dimensional block diagram images representing vertical and horizontal sections of the bedforms (Fig. 1) along with polar plots of bedform migration directions. Rubin and Carter^26^ subsequently revised and rewrote the code in MATLAB and published scripts that, in addition to the block diagrams, generate outputs with 3D bedform topography as well as animation sequences that illustrate bedform deposition over time. As illustrated in Table 1, bedforms can be grouped based on descriptive aspects such as planform shape (2D, 3D), behavior through time (variable, invariable) and crest orientation (transverse, oblique, longitudinal). Even though 3D illustrations (digital images) could still be generated with the updated codes, they do not produce 3D geocellular volumes. This presented an opportunity to modify the animation sequence protocol to produce 3D digital models which can then be further manipulated for various computational applications. This provides yet another breakthrough related to Rubin’s formative work, by allowing broad application of realistic 3D sedimentologic geocellular models tied to well-documented geologic understanding for exploring a wide range of traditional research topics in sedimentary geology and fluid flow simulation.

**Table 1:** Bedform classification criteria used in Rubin and Carter^26^ for the six BAMs presented in Fig. 1.

	Planform Shape			Behavior Through Time		Crest Orientation		
	2D	3D		variable	invariable	transverse	oblique	longitudinal
		sinuous	superimposed					
*# 5*	X				X	X	X	
*# 13*	X			X		X	X	X
*# 27*	X			X		X	X	X
*# 36*		X			X	X		
*# 42a*		X			X		X	
*# 63*			X	X		X		

## Results and Discussion

One advantage of characterizing small scale heterogeneity through the set of codes derived from Rubin’s work is that it encourages users to understand how input parameters affect the final model structure. This presents an opportunity to more closely tie descriptive understanding of clastic sediments and their depositional environments with physical properties and fluid flow performance.

Geostatistical techniques are unlikely to be replaced by the methods presented here, but rather they can be further integrated into the models presented. For example, the binary model output representing the depositional laminae can be used as a training image or conditioning fabric for other stochastic approaches, such as multiple point statistics (MPS)^28^. Such enhancements are likely to produce even more realistic models than those generated here, potentially including normal or inverse grading of bed sediment.

We have not yet explored a variety of model modifications that might be pursued. For example, the digital model populated with petrophysical parameters (e.g. permeability) could be smoothed to provide a more continuous property distribution (as opposed to random sampling from a PDF). This would provide more locally correlated values that are probably more representative of the continuous and smooth changes in properties that are observed in natural specimens^23^. In addition, the incorporation of stochastic noise for laminae position assignment could be employed to modify the strictly periodic representation of current models that result from using sine and cosine functions for generating bedform architectures. Such modifications are likely to present even more geologically-sound representations of depositional facies.

Modification of the Rubin and Carter^26^ codes allows for the generation of realistic digital models of 3D depositional bedform architecture. These models combine a deterministic bedform architecture component mimicking realistic crossbedding geometries with stochastic variability of petrophysical properties. Another advantage of this approach is that it allows consideration of domain sizes larger than the core plugs typically used for laboratory flow experiments, where small sizes may not fully capture depositional architecture. An example of one binary model (BAM #36) with corresponding *P*_*th*_ (threshold capillary pressure) field is provided with the script used to generate it, but the approach is applicable for dozens of previously documented BAMs (and input parameter files) in Rubin^25^. One example application illustrates the use of three different BAMs representing ripple-laminated fluvial sediment for simulating buoyant CO2 migration through *P*_*th*_ field with increasing textural contrast between matrix and laminae, resulting in volumetric fractions of trapped CO_2_ ranging between 2.7% and 69.5% of the model domain.

### Example model

**Figure 2:**
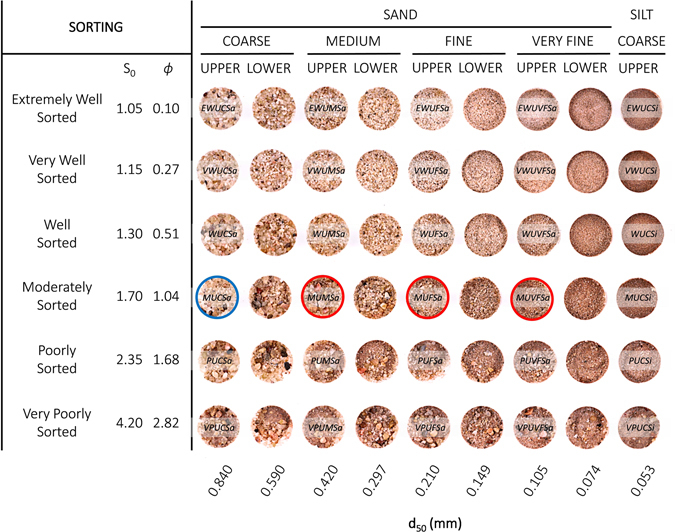
The 54 textural classes used for assignment of matrix and laminae in the models. Each facies has a specific distribution of grain sizes described by a median value (columns) and sorting category (rows). Sorting is given in terms of Trask coefficient *S*_*o*_ and *ϕ* scale^30^. These facies are characteristic of a wide range of depositional processes and environments. For example, very-fine grained, well-sorted silt is typical of upper point bar fluvial deposits; poorly sorted coarse sand is more typical of channel thalwegs. Very well sorted medium sand is typical of shoreface environments. Median grain diameters at the bottom are in millimeters. Permeabilities for these materials range from 471 Darcy (extremely well sorted upper coarse sand, upper left) to 50 milli-Darcy (very poorly sorted upper coarse silt, lower right) (Beard and Weyl^29^, their Table 6). To illustrate the methodology used for generating different textural contrasts, three cases are shown where MUCSa represents the matrix facies and MUMSa, MUFSa, and MUVFSa are used for the laminae (see Fig. 3).

The scripts (m-files) provided enable the user to generate binary digital geocellular models of any of the 79 figures from Rubin and Carter^26^ and to populate each model with any of the facies from Beard and Weyl^29^ (Fig. 2). An example binary model (provided as a MATLAB workspace variable) with a corresponding threshold capillary pressure field that can be generated using the scripts provided is available in the supplementary material (*vert_xsxn_all.mat*). The BAM presented is # 36 of Rubin and Carter^26^, and the block diagram image that is part of the output is shown in Fig. 1. With assigned cell dimensions of 2 × 2 × 2 mm^3^, the model is 90 × 60 × 35 cm^3^. The model matrix is populated with moderately sorted upper coarse sand (MUCSa) and the laminae with moderately sorted upper fine sand (MUFSa), and threshold capillary pressure values are assigned as described above.

### Practical implication

**Figure 3:**
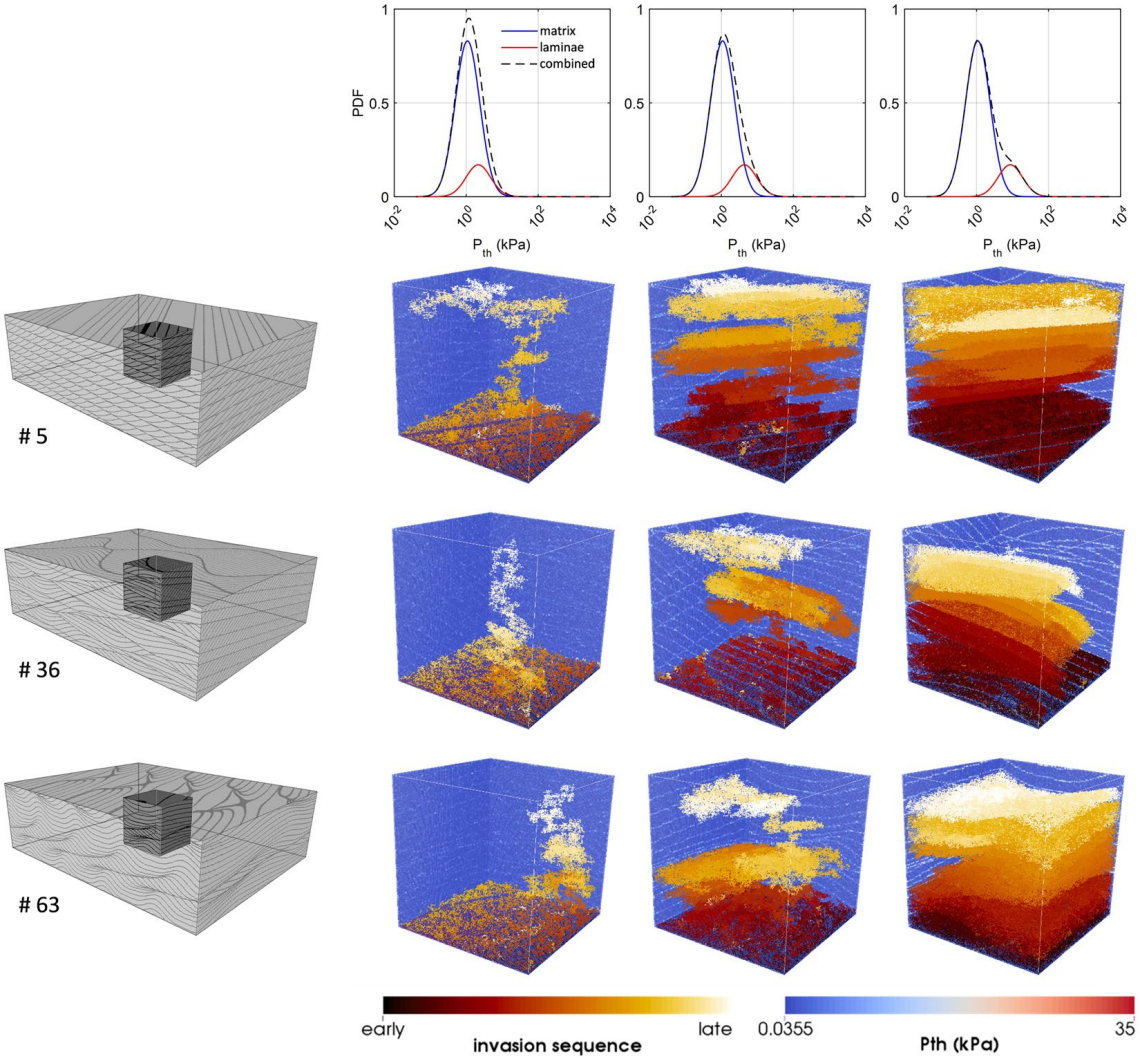
Invasion percolation simulations of CO_2_ migrating through cubic sub-volumes derived from BAMs #5, #36, and #63 (grayscale models on leftmost column) and populated with *P*_*th*_ values generated using three different textural contrasts. The three bimodal distributions (top row) are created by assigning constant *P*_*th*_ PDF to matrix (in this case Moderately Sorted Upper Coarse Sand, or MUCSa, in blue) and changing *P*_*th*_ PDF of laminae (in this case showing Upper Medium Sand > Upper Fine Sand > Upper Very Fine Sand, from low to high *P*_*th*_, in red). Color background of cubic models represents range of threshold capillary pressures (see Fig. 4C). A continuous planar source of CO_2_ was placed at the bottom boundary of the model domain. The increasing textural contrast leads to different responses in terms of displacement patterns and plume trapping as can be observed from invasion sequences of CO_2_.

To provide an example of how the datasets generated can be used, an application is presented illustrating the use of the example model for simulating buoyant fluid migration of supercritical CO_2_ dominated by capillary forces. The movement of carbon dioxide in the subsurface is an important consideration for projects that inject CO_2_ into subsurface reservoirs^31,32^. Far from injection wells, and after injection ceases, buoyancy and capillary forces are known to control flow behaviour, while small scale heterogeneities strongly influence saturation distributions^33^. As a matter of fact, small-scale depositional features have been shown to have significant influence on CO_2_ migration both numerically^5,34–36^ and experimentally^37,38^. Due to the capillary-dominated flow assumptions, the numerical simulations can be carried out in a matter of seconds using the invasion percolation algorithm^39,40^. We use Permedia software to model buoyant migration of CO_2_ from a planar source at the bottom of the model until the CO_2_ plume spans the entire model (i.e., at percolation). The goal of these simulations is to understand the effect of *P*_*th*_ contrast between matrix and laminae as well as the geometry of the crossbedding on the volumetric fraction of trapped CO_2_ plume of each BAM. Figure 3 shows simulation results for three different textural contrasts applied to three different BAMs (#5, #36, and #63). Results from 200 equally-probable realizations of the *P*_*th*_ field (using the same architecture, but different realizations of property assignment) indicate that volumetric fractions of trapped CO_2_ plume in these models is expected to range between 2.7% and 69.5% of the model domain. This high variability provides a realistic expectation for CO_2_ saturations in this type of clastic material for the subsurface conditions considered. This topic is more fully and rigorously explored with more bedform architecture models and facies assignments in Trevisan, *et al*.^41^.

## Methods

### Description of the modified code

The starting point for code development is the MATLAB code by Rubin and Carter^26^. This set of scripts can be used to create images of depositional features with known morphology and interpreted genesis, and can be tied to specific geologic examples that are well documented in the literature^16,25,27^. In the modified scripts provided here, a 3D geocellular model is intuitively built from adjacent 2D vertical sections parallel to a vertical face represented in the block diagrams produced. This is needed because the original script does not explicitly have as part of the output a workspace variable in MATLAB with values representing 3D assignment of matrix and laminae cells.

The primary modification of the original *NewDunes.m* function is a new outer loop that allows a 3D dataset to be generated from adjacent 2D sections (essentially serial slicing vertical planes) of the standard 3D block diagram output. For advanced users who want to understand the procedure which allows a 3D model output, the *for* loop beginning at line 32 of the modified code (*Model_Generator.m*) incrementally changes the input variable PHASEF, which is described by Rubin and Carter^26^ as the bedform phase (in degrees). Changing PHASEF essentially specifies the horizontal position of the bedform within the fixed reference frame of the block diagram edges. By systematically changing this variable and retaining the vertical face of the block diagram output for each loop, serial sections of the model can then be concatenated to build a 3D digital model.

Beyond the addition of simple looping, the modified code takes advantage of a built-in MATLAB function that is perhaps non-intuitive for 3D geologic model building. The modified script uses the ‘vec2mtx’ (line 106 of *Model_Generator.m*) function, which is used in mapping applications and is available through MATLAB’s Mapping Toolbox. More specifically, the ‘vec2mtx’ function converts X-Y vectors (polygons) to a specified regular data grid of binary values (zero or one). Practically, it allows for a continuous representation of depositional laminae in a gridded format, similar to pixelated (raster) representation of a map of political boundaries. This function is applied to each vertical section of the model during the looping of PHASEF described above. This mapping function is needed because the workspace variable representing the laminae on the block diagram faces is initially stored as a three column array representing coordinates of the laminae locations, and is not a regularized grid. For additional information about ‘vec2mtx’, the reader is referred to the MATLAB documentation and numerous online examples.

For users familiar with the routines by Rubin and Carter^26^, the script published here contains comments on each line that represents a modification of the original script or new entry. The new script will produce an image of the model, but all scripting related to visual animation of bedforms in the original script has been eliminated for clarity and compactness. Since the original script contains standard comment notation, new and/or modified lines are identified in the new scripts by the use of four comment characters (%%%%). The comments provide a description of the purpose of the modification or new entry made in each line. As such, the described modifications are fairly minor.

Model domain size (the number of cells in the orthogonal horizontal directions) can be modified by changing the ‘grid density’ variable (line 28 of *Model_Generator.m*) and by defining the area of interest (lines 95, 103 and 105 of *Model_Generator.m*). Higher values of grid density represent the same architecture at increasing resolution, and model sizes of millions of cells are not uncommon for low values of grid density (e.g. 5). The size of the output 3D models is typically assigned considering the wavelengths of the input variables, as defined in the variable input file (e.g. SPCNGF; line 3 of any *Fig##.m* parameter file from Rubin and Carter^26^). This input parameter is defined as the bedform wavelength in the first set, and similar input variables correspond to the second or third bedform set (SPCNGS, SPCNGT, where the final letter corresponds to the set number). Maximum model domain size capability for the modified code was not fully explored, or a variety of other aspects of model generation, as our ultimate goal was standard facies model utilization for flow simulation. The model domain sizes created by the default variables in the figure parameter files of Rubin and Carter^26^ were sufficient for our intended purposes. Other potential (unexplored) modifications are included in the Discussion section.

The output 3D model in the modified scripts does not have prescribed cell physical dimensions. Model dimensions are user-specified after generating the model, and cell dimensions need to be defined by the user in subsequent model applications depending on the knowledge of sedimentary structures, which can be informed by Rubin^25^, examples in literature^42^, and personal experience. Obviously there is subjectivity in cell dimension assignment, but some assignments would produce models with features that are not readily found in natural depositional settings at those scales. That is, sedimentary structures formed by known depositional processes have typical physical dimensions, which should be reflected by reasonable assignment of cell size (and resultant model domain dimensions), based on the user’s knowledge and experience. In the model application presented here, cubic sub-volumes of approximately one million cells with cell dimensions of 0.202 × 0.202 × 0.202 m^3^ are extracted from the 23,625,000-cell model produced by *Model_Generator.m*. The procedure to generate these representative elementary volumes (REV) is explained in appendix A of Trevisan *et al*.^41^.

**Figure 4:**
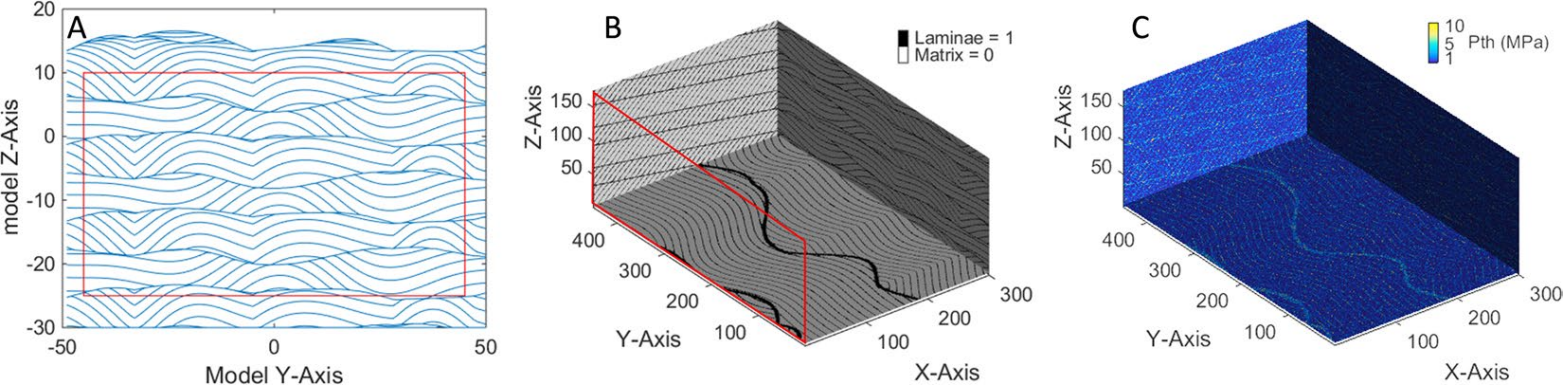
(**A**) Vertical cross section of BAM #36 (see Fig. 1) in the Y-Z plane. The red box represents model subset that is extracted from each vertical slice to build the continuous 3D volume. Horizontal and vertical scale values have no prescribed units, but are reasonably considered to be cm (see text). (**B**) Fence diagram of output binary model showing same architecture of matrix and laminae as in the original Rubin and Carter^26^ graphic output. Red polygon same as in (**A**). Axis units are arbitrary, and differ from (**A**) as a result of the gridding process. (**C**) Sample realization of threshold capillary pressure (*P*_*th*_) values from same model as (**B**) assuming the matrix is moderately sorted upper coarse sand (MUCSa), and the laminae are composed of moderately sorted upper fine sand (MUFSa).

The output of *Model_Generator.m* includes an image of the model domain in the vertical Y-Z plane (Fig. 4A) including the defined area of interest for the model, a fence diagram of the model showing three orthogonal planes (Fig. 4B), as well as a MATLAB workspace variable (“vert_xsxn_all”) with lamina cells represented by ones and matrix cells represented by zeros.

Subsequently, the entirely new script *Value_Assign.m* needs to be run to assign petrophysical properties such as grain sizes and threshold capillary pressure (*P*_*th*_) to the individual model cells (e.g. MATLAB workspace variable named “vert_xsxn_all_trunc_Pth”; Fig. 4C). The assignment of any non-binary physical properties is user defined and can be extended beyond the methods presented here by editing the code, but in the method described here is fundamentally related to specific geologic facies as described below. That method can be adopted, or another preferred method could be employed by the user.

### Property assignment

In addition to generating a synthetic 3D model on a Cartesian grid, the set of codes populates the binary model with petrophysical properties such as grain size, permeability, or threshold capillary pressure. In order to do this, matrix and lamina cells of the binary models are assigned with textural classes characterized by a range of grain sizes and grain sorting.

Beard and Weyl^29^ classified 48 clastic facies of artificially mixed unconsolidated sands into eight sand grain-size subclasses and six sorting groups, whereas Fig. 2 also includes a ninth column with silt. Following previous work of Meckel, *et al*.^43^ we assign the matrix and lamina cells of the output 3D model with the lithofacies shown in Fig. 2. With the purpose of representing realistic depositional environments, grain sizes assigned to matrix facies are always coarser than grain sizes assigned to the laminae. Assignment of identical lithofacies to both laminae and matrix will create a rather homogeneous model, whereas varying amounts of textural contrast between matrix and laminae will regulate the baffle effect exerted by the laminae. For the example application presented, the matrix is considered to be made up of moderately sorted upper coarse sand (MUCSa), while the laminae are composed of, from low to high contrast, moderately sorted upper medium sand (MUMSa), upper fine sand (MUFSa), and upper very fine sand (MUVFSa). The assignment of facies to matrix and laminae is user-defined by screen prompt during execution of the script *Value_Assign.m*. The options for these assignments range from median grain sizes of upper coarse sand to upper coarse silt, and from extremely well sorted to very poorly sorted.

After matrix and lamina facies have been assigned to the binary model, grain size values for each cell are sampled from probability density functions (PDF) corresponding to each lithofacies. The sampling from the probability distribution of grain sizes and corresponding petrophysical properties occurs in the *Value_Assign.m* script via built-in random number generator and is a necessary step to account for the high uncertainty of natural geological heterogeneity. The mean and standard deviation of each PDF are related to the median grain diameter and sorting category, respectively. Each of the facies types shown in Fig. 2 has lognormally distributed grain diameters^44^ and is described by the median diameter (*d*_50_) and the Trask sorting coefficient (*S*_*o*_). The Trask sorting coefficient is defined as the square root of the ratio of the 25^th^ to the 75^th^ percentile of the cumulative density function of the lognormal distribution:

1 $${{\rm{S}}}_{0}=\sqrt{\frac{{{\rm{d}}}_{25}}{{{\rm{d}}}_{75}}}$$

where *d* is grain diameter.

Since the grain sizes are often log normally distributed^44^, the logarithm of the grain size (*D*) will be normally distributed and is given as:

2 $$D=log(d)$$

Then, substituting 2 into 1, the Trask sorting coefficient can be re-written as:

3 $${S}_{0}={e}^{\frac{{D}_{25}-{D}_{75}}{2}}$$

For a normal distribution, the quantile function is defined as:

4 $$D=\mu +\sigma \sqrt{2}er{f}^{-1}(1-2p)$$

where *µ* is the mean, *σ* is the standard deviation, *erf* is the error function, and *p* is the cumulative probability.

The mean of the normal distribution is given by the logarithm of the median of the lognormal distribution:

5 $$\mu =log({d}_{50})$$

Substituting 4 and 5 into 3, we solve for the standard deviation *σ* of the normal distribution. Then, using the calculated mean and standard deviation, we calculate the inverse CDF for cumulative probabilities from 0 to 0.999, which gives the values of the normal distribution. Since these are logarithmic values, the antilog gives back the lognormally distributed grain diameters. These values are then substituted in equation 6 to obtain lognormally distributed *P*_*th*_ values. The lognormal distribution of grain diameters is thus reverse constructed only by using the median and standard deviation values of each of the facies reported in the original paper by Beard and Weyl^29^.

However, if used without any geological knowledge, the results can assign the model cells unnaturally (and irrationally, when considering associated depositional flows) large or small grain size values (outliers in the long tails of lognormal distributions) with subsequent impact on other calculated petrophysical properties (e.g. permeability). So careful scrutiny of model properties is necessary while assigning values from the lognormal distribution.

Beard and Weyl^29^ prepared facies samples by combining sand grains of different sizes such that they were lognormally distributed. But they report that the sand grains used ranged from 0.4 mm to 9 mm in diameter. The same size limits are thus here enforced while populating the models with the codes presented. This is implemented in the program by truncating the distribution between those two grain diameters whenever necessary. The truncation comes into effect only when the distribution is very wide (poorly and very poorly sorted). The code has a provision to change this as per user’s requirements, if there is a preferred method for assigning such properties.

Any grain size distribution obtained in the previous section can be used to calculate equivalent petrophysical parameters like porosity, permeability and capillary entry pressures. For the specific application presented below (i.e. capillary-dominated fluid migration) we populate the model with *P*_*th*_ values.

The capillary entry pressure *P*_*th*_ (in kPa) is obtained from the grain size using equation 6 from Berg^45^:

6 $${P}_{th}=16.3\times \frac{\gamma }{{d}_{50}}$$

where 16.3 is a geometric constant, *γ* is interfacial tension in N/m, and *d*_50_ is median grain diameter, in millimeters. For the example simulation provided we consider 0.03 N/m for CO_2_-H_2_O system, representative for reservoirs at approximately 10 MPa and 35 °C^46^ (~1.5 km depth). These properties are relevant for geological carbon sequestration.

### Data availability statement

All the required MATLAB scripts and one example output dataset generated during the current study are included in this published article (and its Supplementary Information files).

## Electronic supplementary material

Supplementary Information

Supplementary Information

Supplementary Information
