# Author Correction: A method to generate small-scale, high-resolution sedimentary bedform architecture models representing realistic geologic facies

**DOI:** 10.1038/s41598-018-21903-y

**Published:** 2018-03-06

**Authors:** T. A. Meckel, L. Trevisan, P. G. Krishnamurthy

**Affiliations:** 1 Bureau of Economic Geology, Jackson School of Geosciences, The University of Texas at Austin, Austin, TX USA; 2 Department of Petroleum and Geosystems Engineering, The University of Texas at Austin, Austin, TX USA

Correction to: *Scientific Reports* 10.1038/s41598-017-09065-9, published online 23 August 2017

The supplementary datasets were omitted from the original version of this Article. This has now been corrected in the PDF and HTML versions of the Article.

