# Supplementary material for: A method to generate small-scale, high-resolution sedimentary bedform architecture models representing realistic geologic facies
Source: Sci Rep. 2017 Aug 23;7:9238. doi: 10.1038/s41598-017-09065-9 (PMC5569078; doi:10.1038/s41598-017-09065-9)
Supplement: Supplementary file 1 — Supplementary Information [file 41598_2017_9065_MOESM1_ESM.docx]

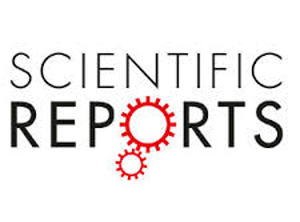


| **Correction – Handover** | | **Author Correction** | | | | |
| --- | --- | --- | --- | --- | --- | --- |
| Original manuscript number: | | SREP-17-26365 | | | | |
| Original DOI: | | 10.1038/s41598-017-09065-9 | | | | |
| Original vol & article number: | | **7:** 9238 | | | | |
| Title of correction: | | Author Correction: A method to generate small-scale, high-resolution sedimentary bedform architecture models representing realistic geologic facies | | | | |
| (Publisher Correction = Erratum, Author Correction = Corrigendum) | | | | | | |
| Corresponding Author: | | | Timothy | Meckel | |  |
| Corresponding Author Email: | | | tip.meckel@beg.utexas.edu | | | |
| Handling Staff: | | | Liam Dickson | | | |
| ‘Non-Fix (standard) or ‘Fix error’ (error fixed in original article)? | | | Fix Error | | | |
|  |  | | | |  |  |
| Text for correction: | | | | | | |
| The supplementary datasets were omitted from the original version of this Article. This has now been corrected in the PDF and HTML versions of the Article. | | | | | | |
|  | | | | | | |
| Notes for typesetting correction: | | | | | | |
|  | | | | | | |

| Changes required to original Article (for production/MPS) |
| --- |
| Please upload attached Supplementary Information files.  Please include the following sentence in the PDF version:  **Supplementary information** accompanies this paper at doi:10.1038/s41598-017-09065-9 |

| Text for Change History section [delete as appropriate] |
| --- |
| A correction to this article has been published and is linked from the HTML version of this paper. The error has been fixed in the paper. |

| Additional Files supplied with Correction | |
| --- | --- |
| Figure |  |
| Figure legend |  |
| Supplementary Information | 2 |

| Error location | Backmatter (Ackn., AC, CFI, Additional Inf) |
| --- | --- |
| Error reason | Author/editor correction belated |
| Who caused the error | Author |
| Affected formats | HTML  PDF  SI |

Additional error details (delete as appropriate):
